# Genome-Wide Identification, Classification, Characterization, and Expression Analysis of the Wall-Associated Kinase Family during Fruit Development and under Wound Stress in Tomato (*Solanum lycopersicum* L.)

**DOI:** 10.3390/genes11101186

**Published:** 2020-10-12

**Authors:** Zongyan Sun, Yanping Song, Di Chen, Yudi Zang, Qiaoli Zhang, Yuetong Yi, Guiqin Qu

**Affiliations:** College of Food Science and Nutritional Engineering, China Agricultural University, Beijing 100083, China; b20173060472@cau.edu.cn (Z.S.); s20193060896@cau.edu.cn (Y.S.); b20193060533@cau.edu.cn (D.C.); zyd3503@163.com (Y.Z.); s20193060911@cau.edu.cn (Q.Z.); 18246076976@163.com (Y.Y.)

**Keywords:** *Solanum lycopersicum*, *SlWAK-RLKs*, phylogenetic analysis, wounding, fruit expression pattern

## Abstract

The wall-associated kinase (WAK) and wall-associated kinase like (WAKL) is a subfamily of receptor-like kinases associated with the cell wall, which have been suggested as sensors of the extracellular environment and triggers of intracellular signals. However, these proteins have not yet been comprehensively analyzed in tomato (*Solanum lycopersicum* L.). In this study, 11 *SlWAK* and 18 *SlWAKL* genes were identified in an uneven distribution in 9 of 12 chromosomes. GUB-WAK-bind (wall-associated receptor kinase galacturonan-binding) and epidermal growth factor (EGF) domains appear more often in SlWAK proteins. However, more SlWAKLs (wall-associated kinase like) have a WAK-assoc (wall-associated receptor kinase C-terminal) domain. Based on their phylogenetic relationships, 29 SlWAK-RLKs (wall associated kinase-receptor like kinases) were clustered into three distinct categories analogous to those in *Arabidopsis thaliana*. High similarities were found in conserved motifs of the genes within each group. Cis-elements in the promoter region of these 29 genes were found mainly in response to methyl jasmonate (MeJA), abscisic acid (ABA), salicylic acid (SA), anaerobic, light, wound, and MYB transcription factors. Public tomato genome RNA-seq data indicates that multiple *SlWAK-RLKs* showed different expression patterns under developmental and ripening stages of fruits, such as *SlWAK4, SlWAKL11, SlWAKL9, SlWAKL15, SlWAKL14*, and *SlWAKL1,* their RPKM (Reads Per Kilo bases per Million reads) value constantly increases during the fruit expansion period, and decreases as the fruit matures. In tomato leaves, our RNA-seq data showed that nine *SlWAK-RLKs* transcripts (*SlWAK3*, *SlWAK4, SlWAK10,* *SlWAKL1, SlWAKL2, SlWAKL3, SlWAKL5, SlWAKL14*, and *SlWAKL18*) were significantly induced (*p* < 0.001), and three transcripts (*SlWAK2*, *SlWAK5*, and *SlWAKL15*) were significantly inhibited (*p* < 0.001) under mechanical wounding. The qRT-PCR (Quantitative reverse transcription polymerase chain reaction) of *SlWAKL1* and *SlWAKL6* verify these results.

## 1. Introduction

Plants are constantly challenged by various biotic and abiotic stresses, causing severe dehydration of plant cell and irreversible damage, which causes substantial losses in the yield and quality of a crop [1]. To survive and multiply in the changing environment, plants have formed a sophisticated and intricate signal transmission network to regulate their own growth and development. Transmembrane sensing signaling proteins are the main regulators in this signaling pathway. They can sense extracellular signals and pass them into intracellular signal-responsive molecules to make plants respond accordingly. There is a class of proteins on the cell surface that sense and transmit signals in animals, called receptor tyrosine kinases (RTKs). Although the molecular structure and function of transmembrane signaling proteins in plants are similar to those of animal RTKs, the extracellular signaling molecules or ligands that specifically bind to them have not been identified, so they are called receptor-like protein kinases (RLKs). RLKs are ubiquitous and constitute a large gene family in many plants with >600 members in Arabidopsis and 1130 in rice, constituting 60% of all kinases and accounting for nearly all transmembrane kinases in Arabidopsis [2,3]. As receptors for signaling molecules, they can sense external environmental stimuli, participate in intracellular signal transmission processes and play important roles in plant growth, development, and communication between plants and environment [4].

Plant RLKs with a clear receptor configuration possess a diverse array of extracellular regions implicated in interactions with proteins, polysaccharides, lipids, and other ligands [3]. According to the difference of extracellular regions, transmembrane receptor kinases can be divided into 15 subfamilies [5]. Wall-associated kinases (WAKs) is one subfamily of the RLKs, containing an extracellular domain with several epidermal factor-like repeats, a transmembrane domain, and one intracellular serine/threonine kinase domain [6,7]. Existing studies have found that *WAK-RLKs* participate in plant cell expansion, development, and stress response. Previous research found that loss of function alleles of individual *WAKs* provided no phenotype in Arabidopsis [8]. However, when the dexamethasone (DEX) induction system was used to construct antisense RNA plant material, the content of WAK protein in Arabidopsis was reduced by 50%, and this led to dwarf plants. Compared with the wide type, leaves of the antisense Arabidopsis showed a smaller cell size, rather than fewer cells [6,9,10], suggesting that WAKs may be an important protein required for cell elongation. In addition, Kohorn et al. [11] later identified a more subtle phenotype for a *WAK2* null allele, *wak2-1*, that caused a loss of cell expansion in roots under limiting sugar and salt conditions [8].

Previous studies found that the expression of *WAKs* can be induced under many biotic and abiotic stress, such as pathogen infection, injury, ozone and heavy metals [7,12,13]. In Arabidopsis, He et al. used plant material lacking the extracellular domain of *AtWAK1* and anti-sense RNA material of *AtWAK1* to directly prove that WAK was involved in plant stress responses, and both of these plant materials showed extremely strong resistance to salicylic acid [12]. In maize, *ZmWAK* was found to be an important gene for resistance to head smut. Corn resistance to head smut was controlled by multiple quantitative trait loci (QTL), and a QTL of corn silk smut called qHSR1 was detected in the bni2.09 region of the maize chromosome. *ZmWAK* is located in qHSR1, which confers quantitative resistance to corn smut [14]. In addition, using high-throughput map-based cloning, it was discovered that the maize leaf spot resistance gene *Htn1* actually encoded a WAK-like protein named ZmWAK-RLK1; it confers quantitative resistance to northern corn leaf blight (NCLB) by inhibiting the biosynthesis of secondary metabolites, benzoxazinoids (BXs), which suppress pathogen penetration into host tissues [15,16]. In rice, *OsWAK1*, shown to be induced by wounding, SA, methyl-jasmonate, and rice blast (*M. oryzae*), but not abscisic acid. Furthermore, overexpression of *OsWAK1* conferred resistance to *M. oryzae* [17]. In another study, a reporter construct containing the *OsWAK11* promoter fused to GUS is activated in response to wounding [18]. In tomato, Zhang et al. generated two homozygous *wak1* mutant lines using CRISPR/Cas9, and found that *Wak1* protein acted as an important positive regulator in later stages of flagellin-mediated PTI (PAMP-triggered immunity) response in the apoplast and formed a complex with Fls2 and Fls3 to trigger immune signaling [19].Genome sequences can provide valuable information for genome-based investigation, such as gene cloning and genome-wide analysis of a target gene family [20]. *WAK-RLKs* had been investigated using global identification techniques and genome analysis in some plants. To date, 26 *WAK-RLKs* in Arabidopsis, 44 *WAK-RLKs* in apple [21], 130 *WAK-RLKs* from *O. sativa japonica*, and 111 *WAK-RLKs* from *indica* [22] had been identified. However, as the principal model to study fleshy fruit development, ripening, and biotic or abiotic stress response, relatively little information is available about the identification, structure, organization, phylogeny, and expression profiles of *WAK-RLK* gene family members in tomato, and less about their responses during growth and development or in response to the mechanical wounding. Here, we presented identification and characterization of tomato WAK-RLK gene family members comprised of 29 genes in the tomato genome. A detailed in silico study for WAK-RLK proteins for characteristic protein domains, their localization, gene duplication events, gene structure and phylogeny were presented. We also analyzed the transcript accumulation of each of the family members utilizing a combined transcriptome and qRT-PCR approach, which provided a comprehensive insight into differential transcript accumulation patterns during fruit development and ripening. Further, we demonstrated the response patterns of each tomato *WAK-RLK* gene family member to mechanical wounding. This study aimed to provide a comprehensive view of the *WAK-RLK* genes in tomato and to identify members involved in the development and ripening of tomato as well as the wounding response.

## 2. Materials and Methods

### 2.1. Plant Materials and Wounding Treatment

Tomato (*Solanum lycopersicum* L.) cv. Micro-Tom plants were grown in a glasshouse with a 16 h light/8 h dark cycle at 25 °C and cv. Ailsa Craig (AC) plants were grown in a glasshouse with natural light and temperature.

For leaves wounding treatment, mature and fully stretched Micro-Tom tomato leaves from 28 days old plants were wounded with a hemostat across the midrib of all leaflets (Supplementary Figure S3) [23]. Wounded plants were incubated under continuous light conditions. At each sampling time point, six whole plants leaves were harvested as one replicate for extracting total RNA and each of the experiment with three replicates. Control leaf tissue was collected from wounded plant at 0 h.

AC fruits at mature green stage were used for wounding treatment. Before the treatment, fruits were washed with 75% ethanol and let dry in an ultra-clean table. All procedures were performed under sanitized conditions to avoid microbial growth. Pericarp discs were excised from the equatorial area of the fruit using a 10 mm hole punch and placed with endocarp down in the petri dishes with a sterile water-soaked filter paper. Petri dishes were closed and placed at 22 °C [24]. Six discs were used for each replicate and each of the experiment consisted of three replicates.

### 2.2. SlWAK-RLKs Identification

The Hidden Markov Model (HMM) files corresponding to the EGF (PF00008) and pkinase (PF00069) domains were download from the Pfam protein family database (http://pfam.xfam.org/), TBtools was used to search against the WAK genes from the *Solanum lycopersicum* L. genomic database SL4.0 (https://solgenomics.net/organism/Solanum_lycopersicum/genome/) [21,25]. As a result, only 15 genes were identified (Supplementary Table S10). To get more WAK-RLK members, feature sequences of RLK-Pelle WAK and RLK-Pelle_WAK_LRK10L-1 (Supplementary Table S2) were used to query and scan the protein database with BLASTp, with an E-value cut-off ≤ 1.93 × 10^−22^. These two sequences were downloaded from iTAK (http://itak.feilab.net/cgi-bin/itak/index.cgi), which had classified plant protein kinases by comparing their sequences to a set of Hidden Markov Models (HMMs) developed by Lehti-Shiu [26]. All sequences on the BLASTp alignment were comprehensively sorted according to E-value and score. We performed protein domain analysis through SMART (Simple Modular Architecture Research Tool) (http://smart.embl-heidelberg.de/) [27] and CDD (Conserved Domain Database) (https://www.ncbi.nlm.nih.gov/Structure/cdd/wrpsb.cgi) [28] to verify the protein structure in turn. Members that only contain the pkinase domain without any other WAK conserved signature domains (EGF, EGF-CA, TM, and GUB-WAK) were removed. Finally, 29 SlWAK-RLKs family members were identified, including 11 SlWAKs and 18 SlWAKLs.

### 2.3. SlWAK-RLKs Sequence Analysis

The Physicochemical characteristic of SlWAK-RLKs, including protein length, isoelectric point (pI) and molecular weight (MW) were predicted by ExPASy ProtParam (http://web.expasy.org/protparam/) [29,30]. SlWAK-RLKs protein structure prediction information were obtained from the SMART website (http://smart.embl-heidelberg.de/) [21,27]. Multiple sequence alignments of 29 *SlWAK-RLKs* were conducted with Clustal Omega online (https://www.ebi.ac.uk/Tools/msa/clustalo/) [31] and edited with Jalview 2.11.0. All annotations and drawings of domain information, including GUB-WAK-bind, EGF-CA, WAK-assoc, WAK, Pinase, Signal peptide and Transmembrane domain were derived from SMART.

### 2.4. Phylogenetic Analysis

The phylogenetic analysis was performed with 29 SlWAK proteins (Supplementary Tables S1–S3) from *Solanum lycopersicum* L., and 26 AtWAK proteins (Supplementary Table S5) from *A. thaliana* [32]. The phylogenetic tree was constructed using MEGA-X software (www.megasoftware.net) [33] with the neighbor-joining (NJ) method and was analyzed with a poisson-correction substitution model with 1000 bootstrap replications [21,34]. According to the results of phylogenetic analysis, WAKs were divided into different groups, and marked with different colors.

### 2.5. Gene Structure, Conserved Motif and Chromosome Location Analysis

The Gene Structure Display Server (http://gsds.cbi.pku.edu.cn) is a tool to display the exon-intron organizations of a gene. The annotation of *SlWAK-RLKs* displayed in the NCBI were used to performed the analysis (https://www.ncbi.nlm.nih.gov/) [21,35].

The MEME (http://meme-suite.org/) is the tool used to search the motifs of *SlWAK-RLKs* [36]. The parameters were set as follows: the site distribution was set to any number of repetitions, the number was set to 10, the width was limited to between 6 and 50; and other optional parameters remained default [37]. The chromosomal location of genes of *SlWAK-RLKs* were maped by MapGene2Chrom web v2 online (http://mg2c.iask.in/mg2c_v2.1/) [38].

### 2.6. Cis-Element Prediction for SlWAK-RLK Gene Promoter

To search for cis-acting elements in the promoter sequences of *SlWAK-RLKs*, genomic DNA sequences in the promoter region (−1500 bp to −1 bp) were downloaded from NCBI and were subsequently scanned using the Plant CARE database (https://www.ncbi.nlm.nih.gov/;be/webtools/plantcare/html/) [21,39].

### 2.7. Heat Map Analysis

RNA-seq data of various tomato tissues [20], including 1, 2, 3 cm fruit, mature green fruit, breaker fruit, and fruit at 10 days after break, were analyzed. Excluding the RPKM 0 in any organs and stage. Relative expression values were transformed to log2 (RPKM) to represent relative expression levels.

### 2.8. RNA Isolation and qRT-PCR Analysis

Total RNA was isolated from each sample using a total RNA isolation kit (OMEGA, Norcross, GA, USA). Reverse transcription was performed using a PrimeScript One-Step gDNA Removal and cDNA Synthesis Super Mix (TransGen Biotech, Beijing, China). Quantitative RT-PCR was performed on a CFX96 Real-Time PCR (Bio-Rad, Berkeley, CA, USA) with TransStart Top Green qPCR SuperMix (TransGen Biotech, Beijing, China) using qRT-PCR primers (Supplementary Table S9), with the *Actin* transcripts as internal control.

### 2.9. RNA Sequencing and Bioinformatics Analysis

Total RNA was extracted from tomato wound 0 h (w-0 h) and 1 h (w-1 h) leaves using a total RNA isolation kit (OMEGA, Norcross, GA, USA), three biological replicates were made for each sample. The mRNA was enriched using oligo-dTs coupled with magnetic beads before being cut into 300 bp fragments (Novogene, Beijing, China). Next, RNA-seq libraries were sequenced on HiSeq PE150 (Illumina, San Diego, CA, USA) with approximately 14 million 150-bp paired-end reads. Clean reads were quality checked using FastQC (version 0.11.3). Reads were aligned to the tomato reference genome (version SL3.0) using Tophat (version 1.4.6), and fragments were assigned to genes using Feature Counts (version 2.0.14) [40,41]. Gene expression abundance was represented by RPKM value. Differences in gene expression between w-0 h and w-1 h leaves were identified by DESeq2 Library [42]. The fold change was calculated by RPKMw-1h/RPKMw-0h. Genes were considered as differentially expressed genes (DEGs) between w-0 h and w-1 h if |log2 (FoldChange)| > 1 and *p*-value < 0.001.

## 3. Results

### 3.1. Genome-Wide Identification of SlWAK-RLK Genes in Tomato

To characterize putative Wall-Associated Kinases in tomato, 11 SlWAK (Supplementary Table S3) and 18 SlWAKL (Supplementary Table S4) proteins with typical Kinase domain were obtained based on the BLASTp search against the tomato genome database (available online: http://solgenomics.net/). The detailed information of SlWAK-RLKs, including gene name, Soly ID, chromosome location, length of protein, theoretical isoelectric point, and molecular weight are presented in Table 1 and Supplementary Table S1. The length of SlWAK-RLKs differed from 302 (SlWAKL13) to 799 (SlWAK4) with an average length of 630 amino acids (aa). Molecular weight varied from 33.7 kDa (SlWAKL4) to 88.3 kDa (SlWAK4), and the isoelectric point varied from 5.39 (SlWAKL4) to 8.85 (SlWAKL16), with an average of 6.73, showing a weakly acidic. The location of these 29 genes distributed on 9 (Excluding chromosome 1, 6, and 8) of the 12 tomato chromosomes, with most being located on the 5th and 9th chromosomes.

### 3.2. Conserved Motif and Evolutionary Analysis of SlWAK-RLK Proteins

**Figure 1:**
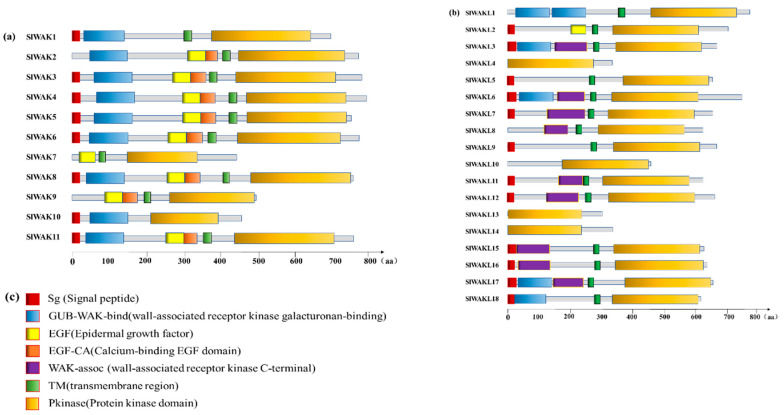
Structure of the tomato SlWAK-RLK proteins. Different structural domains are represented by different color blocks, the specific details have been marked in the figure. Scale bar represents 800 amino acids. Domains were identified using SMART (http://smart.embl-heidelberg.de/). (**a**) Structure of the tomato SlWAK proteins; (**b**) Structure of the tomato SlWAKL proteins; (**c**) The domains found in these SlWAK-RLK proteins.

**Figure 2:**
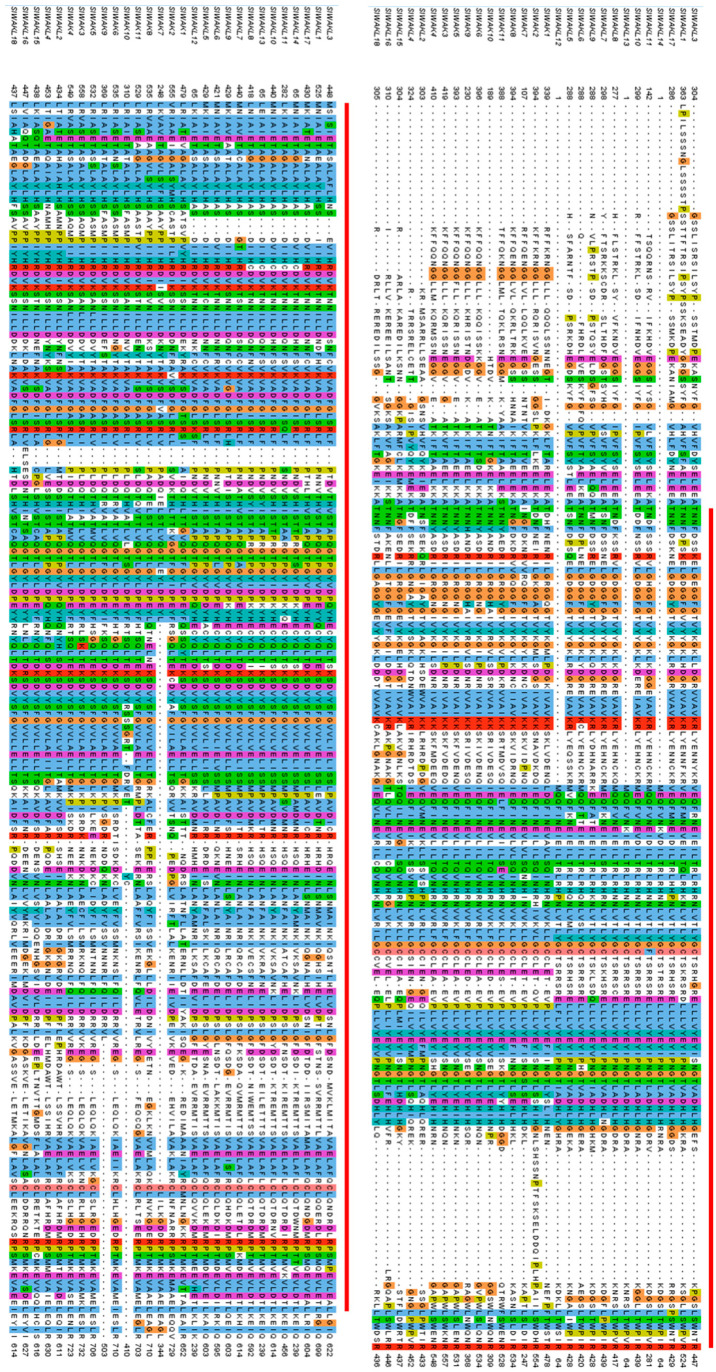
Sequence alignment of all identified SlWAK-RLK proteins. The SlWAK-RLKs family signature Pkinase in tomato is underlined. The conserved residues are indicated by a colorful background.

In animals, RTK exhibited great diversity in their extracellular regions, but shared a highly conserved intracellular tyrosine kinase domain [43], and the RLKs in plants have similar structure. For tomato SlWAK-RLKs, SMART analysis indicated that specific domains exist in the extracellular domain of SlWAKs and SlWAKLs respectively (Figure 1). Nine out of 11 SlWAKs (Excluding SlWAK7 and SlWAK9) proteins have the GUB-WAK-bind (wall-associated receptor kinase galacturonan-binding, marked by blue square) domain; 9 out of 11 SlWAKs (Excluding SlWAK1 and SlWAK10) have the EGF (Epidermal growth factor, marked by yellow square) followed by a EGF-CA (Calcium-binding EGF domain, marked by orange square). In contrast, for SlWAKLs, GUB-WAK-bind and EGF domains rarely appear. However, a WAK-assoc (wall-associated receptor kinase C-terminal, marked by purple square) domain especially exists in the extracellular domains of multiple SlWAKLs. In addition, signal peptides and transmembrane domains appear in SlWAKs and SlWAKLs without distinction. A total of 20 out of 29 SlWAK-RLK proteins contain a N-terminal signal peptide located at extracellular domain; 24 out of 29 SlWAK-RLK proteins possess the TM domain. All SlWAK-RLK proteins contain a conserved intracellular domain named Pkinase, and sequence alignment confirmed this result (Figure 2 and Supplementary Figure S1).

**Figure 3:**
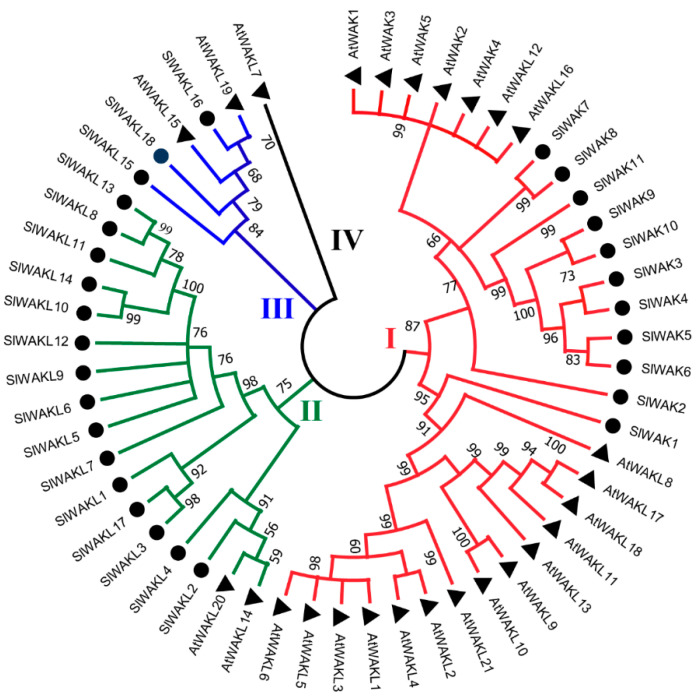
Phylogenetic analysis of WAK-RLKs. The phylogenetic tree was generated using the amino acid sequences of selected WAK-RLKs via NJ methods. All tomato WAK-RLKs, together with theirArabidopsis thaliana homologues were classified into 4 groups. At represented *A. thaliana*.

To explore the evolutionary relationship of WAK-RLK family, the WAK-RLK protein sequences of *Solanum lycopersicum L.* and *A. thaliana* were used to construct the phylogenetic tree. Total 29 SlWAK-RLKs and 26 AtWAK-RLKs were clearly divided into 4 groups, here named as Groups I to IV, and Group IV only contains one protein AtWAKL7 (Figure 3). SlWAK-RLKs distribute unevenly in the 3 groups including Group I, II and III. Eleven SlWAKs were fully assigned to Group I, meanwhile, 21 AtWAK-RLKs belonged to this group. These results means that SlWAKs have a closer evolutionary relationship with AtWAK-RLKs compared to SlWAKLs. Group II included 15 SlWAKLs and 2 AtWAKLS, Group III was composed of 2 SlWAKLs and 3 AtWAKLs members. Previous research on WAK-RLKs are mainly concentrated in *A. thaliana*, and the understanding of the evolutionary relationship between SlWAK and AtWAK provides guidance for the further research of SlWAK-RLKs.

### 3.3. Chromosome Location, Gene Structure, and Motif Patterns of SlWAK-RLKs

**Figure 4:**
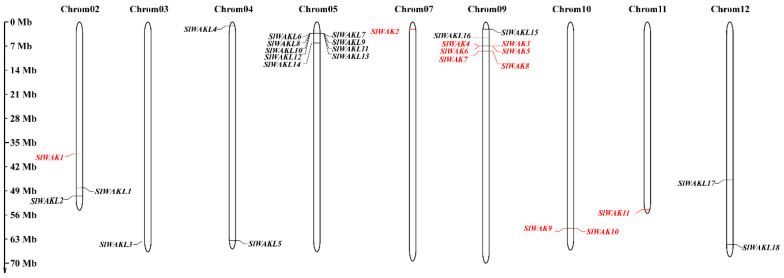
Chromosome distribution and duplication events of tomato *SlWAK-RLK* genes. Chromosome localization is based on the physical location (Mb) of 12 tomato chromosomes. Chromosome numbers are displayed at the top of each bar chart. The red indicates the *SlWAKs*, and the black represents the *SlWAKLs*. The locations of tomato *SlWAK-RLK* genes in chromosomes were obtained from the Sol Genomics Network database (http://solgenomics.net). Scale represents 70 Mb chromosomal distance.

To examine the chromosomal distribution of the *SlWAK-RLKs*, Genomic positions of them were obtained from the *Solanaceae lycopersicum* gene model ITAG4.0 and were mapped onto the corresponding tomato chromosome locations. Notably, these *SlWAK-RLKs* were unevenly distributed on nine chromosomes of 12 total chromosomes (Figure 4). High-density regions harboring *SlWAK-RLKs* were discovered in chromosomes 5 and 9 and existed in the form of gene clusters as shown in Table 1. Meanwhile, the members were not randomly distributed, and most members were positionally close when located on the same chromosome. Chromosome 5 contained the most *SlWAKL memebers* and there is a *SlWAKL* gene clusters including 7 *SlWAKL* genes (*SlWAKL6*, *SlWAKL7*, *SlWAKL8*, *SlWAKL9*, *SlWAKL10, SlWAKL11,* and *SlWAKL12*). Chromosome 9 contained the most *SlWAK* genes and there are two *SlWAK* gene clusters, one including four genes (*SlWAK3, SlWAK4, SlWAK5,* and *SlWAK6*), and another including two genes (*SlWAK7* and *SlWAK8*). Only one *SlWAK-RLK* gene on chromosome 3, 7, and 11, respectively, and two SlWAK-RLK genes on chromosome 4, 10, and 12 respectively. Three SlWAK-RLKs mapped onto chromosome 2 and no SlWAK-RLKs was found on chromosomes 1, 6, and 8.

**Figure 5:**
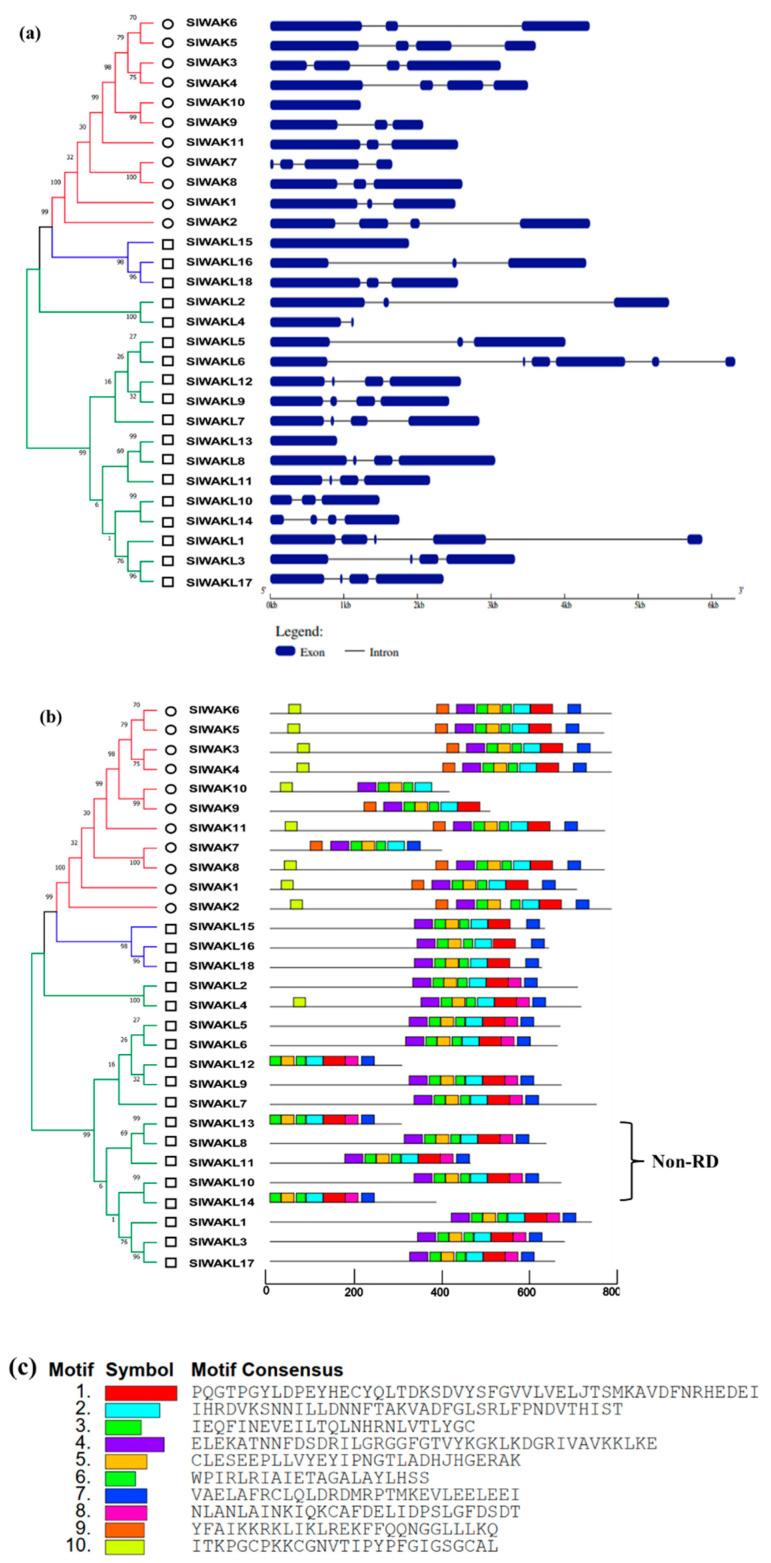
Gene structure and motif patterns of the tomato *SlWAK-RLK* genes. (**a**) Gene structure of the tomato *SlWAK-RLK* genes generated using Gene Structure Display Server (http://gsds.cbi.pku.edu.cn). The yellow block indicates the coding sequence (CDS), and the black line represents the intron. The lengths of the DNA sequences are indicated by the scale bar. The red number in front of the gene ID represents the group to which the gene belongs in the Phylogenetic analysis; (**b**) Conserved motif analysis of the tomato *SlWAK-RLK* genes generated using MEME (http://meme-suite.org/). Black solid line represents corresponding *SlWAK-RLKs* and their size. Various colored boxes indicate different motifs; (**c**) The motif consensus of various colored boxes. Numbers 1–11 represent the grouping of the gene in the evolutionary tree in Figure 3.

To understand the structural diversity of *SlWAK-RLK* genes, the tomato *SlWAK-RLKs* exon–intron organizations were analyzed. As shown in Figure 5a, most of the *SlWAK-RLKs* contained 2 or 3 introns, except for *SlWAK10*, *SlWAKL13*, and *SlWAKL15*, which have no introns; *SlWAKL4* contains one intron; *SlWAKL1*, and *SlWAKL6* have four and five introns, respectively. The results show that there is no obvious difference of gene structures between different groups. However, genes from the same gene cluster have similar gene structures. For example, the gene cluster composed of SlWAKL6, SlWAKL7, SlWAKL8, SlWAKL9, SlWAKL10, SlWAKL11, and SlWAKL12 prefers three introns. These phenomena may be due to the absence or gain of exons during long-term evolutionary processes.

To further reveal the structural diversity and functional characteristics of SlWAK-RLKs, the motif patterns among SlWAK-RLKs were generated using MEME (Figure 5b). A total 10 motifs (Figure 5c) were listed in SlWAK-RLK proteins family which were divided into Group I, II, and III, seven of 10 motifs were generally spread in most family members. The remaining three motifs, 8, 9, and 10, may be used as the specific motif for distinguishing SlWAKs from SlWAKLs. The motif 8 is distributed on all derived from the SlWAKLs in the Group II Ten SlWAK-RLKs have motif 9, and nine out of them are derived from Group I. Motifs from the same gene cluster show high similarity. For example, *SlWAK5*, *SlWAK6, SlWAK7,* and *SlWAK8; SlWAKL6, SlWAKL7, SlWAKL8, SlWAKL9, SlWAKL10, SlWAKL11,* and *SlWAK12* have the same motifs respectively. This phenomenon indicates that genes from the same cluster may share extremely similar functions [19].

Previous study found that most PRR kinases or PRR-associated kinases contain a change in a conserved arginine (R) located adjacent to the key catalytic aspartate (D) (the so-called RD motif) that facilitates phosphor transferring [44]. When we analyzed the RD motif in SlWAK-RLKs, we found SlWAKL8, SlWAKL10, SlWAKL11, SlWAKL13, and SlWAKL14 are Non-RD kinases, and the remaining 24 members are RD kinases (Supplementary Figure S2). Interestingly, these five Non-RD kinases are evolutionarily conservative and locate on the same chromosome. It was concluded that the position in the phylogeny was affected by gene structure and motif patterns.

### 3.4. Putative Cis-Regulatory Elements in the Promoter Regions of SlWAK-RLKs

The cis-regulatory elements are the specific motifs that bound by appropriate transcription factors to regulate gene transcription in plants [45,46]. To gain further insights into the transcriptional regulation and potential functions of *SlWAK-RLKs*, the putative cis-regulatory elements in the 1500 bp upstream region of the initiation codon were analyzed using the PlantCARE database. A total of 57 putative cis-regulatory elements were identified (Supplementary Table S6), and well-defined phytohormone responsive, abiotic stress-responsive, and development-related cis-elements were selected and classified in Table 2. The phytohormone responsive categories have the largest number and widespread distribution, such as MeJA-responsive elements (TGACG-motif and CGTCA-motif), abscisic acid-responsive elements (ABRE), salicylic acid-responsive elements (TCA-element), gibberellin-responsive elements (*p*-box, GARE-motif, and TATC-box), and auxin responsive elements (TGA-element and AuxRR-core). Among these elements, the largest part of motifs are related to the signal pathways of MeJA, ABA, and SA. Since ABA, SA and MeJA are important signaling molecules in plant stress responses [47], it is likely that most of members of the WAK-RLKs are involved in response to biotic and abiotic stresses. Along this, stress response-related cis-elements were found in the promoter region of *SlWAK-RLKs*, such as BOX4 and TCT motif (light response), ARE (anaerobic induction), WUN motif (wound-responsive element), and LTR (low temperature-responsive). In the last category, most of the *SlWAK-RLK* genes possessed MYB binding sites, including MYB and MBS. More cis-elements with undefined functions are shown in Supplementary Table S4. These results indicated that *SlWAK-RLKs* might play important roles in response to hormones and stress signals.

### 3.5. Expression Patterns of SlWAK-RLKs during Tomato Fruit Development and Ripening

**Figure 6:**
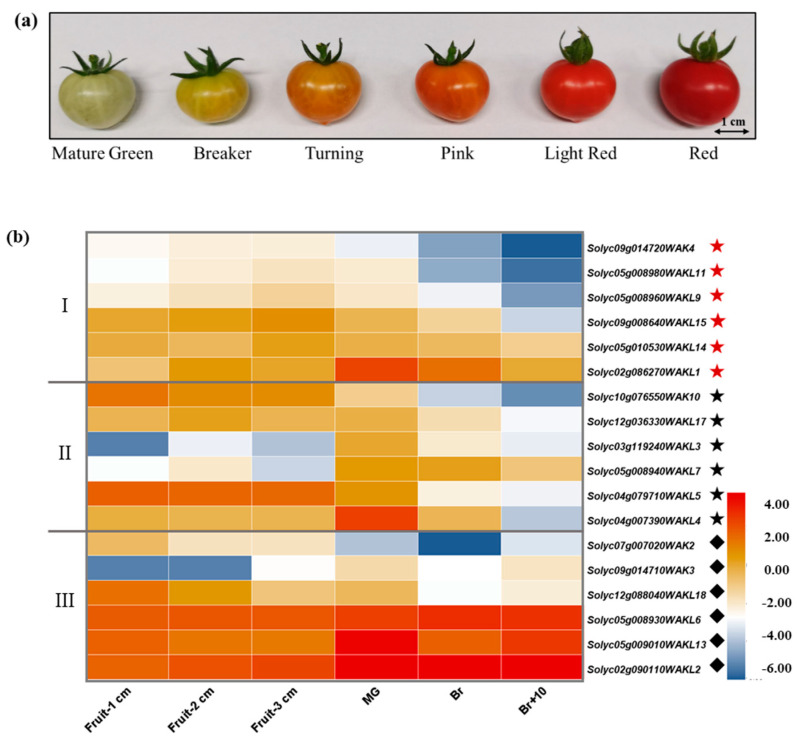
Expression analysis of *SlWAK-RLKs* across different fruit development stages. (**a**) Tomato fruits of different stages used in the experiment; (**b**) Expression analysis of *SlWAK-RLK* genes in various tomato fruit development stages. The RNA-seq expression data (Tomato Genome Consortium, 2012) of various stages were used to reconstruct expression patterns of *SlWAK-RLK* genes in tomato cultivar Heinz. Excluding the RPKM 0 in any organs and stage. Relative expression values were transformed to log_2_(RPKM) represent relative expression levels. MG, mature green stage; B, breaker stage; B10, 10 days post-breaker. Icon on the right side of gene names indicate specific expressed genes.

Transcriptome sequence provides general gene expression patterns of certain gene family members. To understand the putative function of *SlWAK-RLKs* during tomato fruit development and ripening, we firstly analyzed expression levels of tomato *SlWAK-RLK* genes in different fruit development and ripening stage (Fruit 1 cm, Fruit 2 cm, Fruit 3 cm, Mature Green, Breaker stage, and 10 days post-breaker stage) by using public Tomato Genome RNA-seq data (Supplementary Table S7) [20]. After excluding sequences with RPKM = 0, 4 *SlWAKs* (*SlWAK2, SlWAK3, SlWAK4,* and *SlWAK10*) and 14 *SlWAKLs* (Excluding *SlWAKL8, SlWAKL12, SlWAKL13,* and *SlWAKL16*) were selected, indicating that *SlWAKL* genes are widely expressed in tomato genome than that of *SlWAKs*. According to the expression patterns of these genes during tomato fruit development and ripening, they are divided into three categories (Figure 6b, marked with red asterisks, asterisks and diamonds respectively). The six genes in the first group are *SlWAK4*, *SlWAKL11*, *SlWAKL9, SlWAKL15*, *SlWAKL14,* and *SlWAKL1*. Their RPKM valueis constantly increasing during the fruit expansion period, and keep decreasing as the fruit ripening. The second group also contains six genes, including *SlWAK10*, *SlWAKL17*, *SlWAKL3*, *SlWAKL7*, *SlWAKL5,* and *SlWAKL4*. Their RKPM value is not regular in the fruit expansion period, but they are also continuously decreasing during the fruit ripening period. No matter in the expansion stage or the ripening stage of tomato fruit, the expression of the remaining six genes are irregular in the third group. However, the expression levels of *WAKL6*, *WAKL13,* and *WAKL2* are relatively higher than other genes. The above results indicate that multiple *SlWAK-RLK* members may be involved in the development and maturation of tomato fruits.

To verify the expression profile of *SlWAK-RLK* genes in tomato fruits, the fruits of different stages (Mature Green, Breaker, Turning, Pink, Light Red and Red stage, seen as Figure 6a) were selected to detect the expression levels of 6 genes -by qRT-PCR, namely *SlWAK3, SlWAK7*, *SlWAK10*, *SlWAKL1*, *SlWAKL2*, and *SlWAKL6*. As shown in Table 3, during the tomato fruit ripening process, *SlWAKL2* and *SlWAKL6* were more highly expressed, and have the highest expression at pink stage. *SlWAKL7* had low relative expression. The expression of *SlWAK3, SlWAK7,* and *SlWAKL1* decreased during the earlier ripening period. These results are consistent with public Tomato Genome RNA-seq data. Taken together, these results once again suggest that tomato *SlWAK-RLKs* may play roles in the development and maturation of tomato fruits.

### 3.6. Screen and Identified Mechanical Wound Induced SlWAK-RLK Genes

**Figure 7:**
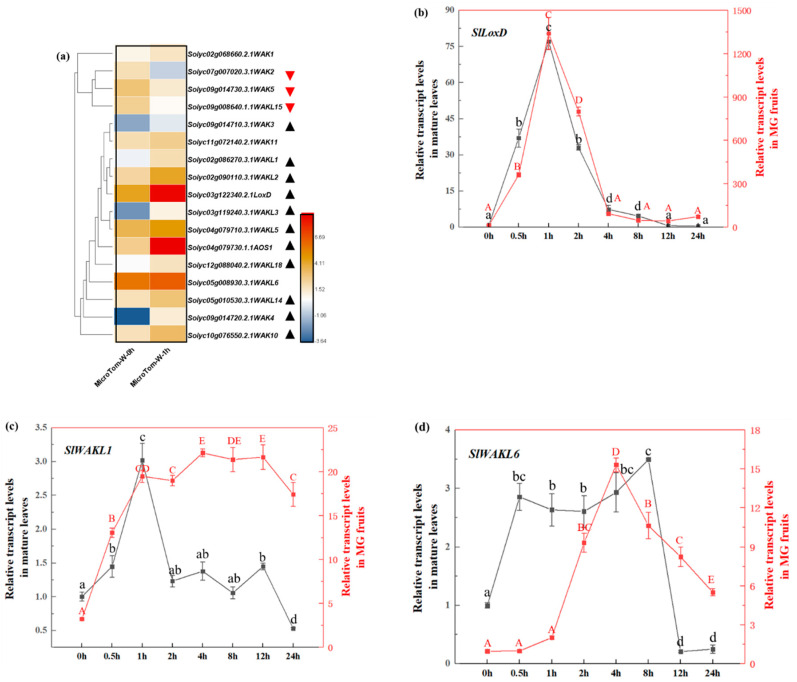
Expression analysis of *SlWAK-RLKs* in response to mechanical wounding. (**a**) Expression analysis of *SlWAK-RLK* genes in wounded mature leaf for 1 h. The RNA-seq expression data of various tissues/stages were used to reconstruct expression patterns of *SlWAK-RLK* genes. Excluding the RPKM 0 in any organs and stage. Relative expression values were transformed to log2(RPKM) represent relative expression levels. ▲ are used to marker the genes upregulated, and ▼ are used to marker the genes downregulated; (**b**–**d**) Time course transcript levels *of SlLoxD*, *SlWAKL1* and *SlWAKL6* in response to mechanical wounding in mature leaves and MG Fruits. The expression levels of the genes in mature leaves at 0 h are set to 1. Data are means ± SD (*n* = 3). Letters above the bars indicate statistically significant difference (*p* < 0.05).

To get general tomato *SlWAK-RLK* genes expression profile after wounding, we performed RNA-seq using hemostat treated leaves from 28 days old plants to screen wounding regulated members. In our sequence data, wounding response marker gene, such as *SlLoxD* (*Solyc03g122340.2.1*) and *SlAOS1* (*Solyc04g079730.1.1*), are significantly increased (*p* < 0.001) compare to that of the control at 1 h after wound (Figure 7a, marked with triangles), indicating our RNA sequencing data are reliable. Then, 29 putative *SlWAK-RLKs* were screened, and 17 of 29 had detectable RPKM value (Supplementary Table S8). After excluding sequences with RPKM = 0, 15 *SlWAK-RLKs* were selected to further analysis, nine genes of *SlWAK3, SlWAK4, SlWAK10*, *SlWAKL1, SlWAKL2, SlWAKL3, SlWAKL5, SlWAKL14,* and *SlWAKL18*, marked with triangles were significantly upregulated to 3.1, 25.1, 2.8, 2.9, 4.0, 9.6, 2.1, 2.3, and 2.4-fold (*p* < 0.001), three genes (*SlWAK2*, *SlWAK5*, and *SlWAKL15*, marked with red inverted triangles) were downregulated to −2.5, Please provide full name.1.6 and Please provide full name.1.8-fold (*p* < 0.001) (Figure 7a and Supplementary Table S8). This RNA-seq data indicates that tomato *SlWAK-RLKs* are mechanical wounding response genes in leaves, and most members are upregulated by wounding.

To further give a dynamic comparation of *SlWAKs* expression in the wounding leaves and fruits, hemostat treated leaves and mature green fruits discs were harvested at indicated time points as Figure 7b–d shown. *SlWAKL1* and *SlWAKL6* were selected to study their expression patterns within 24 h after wounding. The expression of *SlLoxD*, an early wound-inducible marker gene [23], was induced when mature leaves and mature green fruits were mechanically wounded and showed a similar expression tendency (Figure 7b). The levels of *SlWAKL1* transcripts in leaves were induced sharply and significantly (*p* < 0.05) by wounding within 30 min, peaked at 1 h and 3.0-fold (*p* < 0.05) greater than that at 0 h in both leaves and fruits. Subsequently, *SlWAKL1* in leaves showed a sharply decline and returned to control level at 2 h after wounding (Figure 7c). In fruits, the expression of *SlWAKL1* peaked at 1 h significantly (*p* < 0.05), and then maintained about 7-fold (*p* < 0.05) higher than that of control during 24 h (Figure 7c). For *SlWAKL6*, the expression levels of *SlWAKL6* in leaves and fruits are equivalent at 0 h. However, the transcript of *SlWAKL6* in leaves were induced more quickly and peaked at 1 h after wounding, then the expression level remained 3-fold higher until it fell back after 12 h (Figure 7d). In fruits, *SlWAKL6* was induced by wounding within 1h and up to 15-fold at 4 h, then showed a tendency of decline and had not subsided to the control level until 24 h. These results indicated that *SlWAKL1* and *SlWAKL6* are early wound-inducible genes. However, their expression pattern after wounding treatment show to some extent variations in different tissues. When the leaves are damaged, the up-regulation of *SlWAKL1* and *SlWAKL6* is rapid and short-lived, however, the changes in the fruit are slow and persistent.

## 4. Discussion

Wall associated kinases represent a unique class of receptor-like kinase genes that span the plasma membrane and allow cells to recognize and respond to their extracellular environment [48]. In this study, a total of 29 SlWAK-RLKs including 11 SlWAKs and 18 SlWAKLs with typical Kinase domain. In addition, our phylogenetic analysis show all 29 SlWAK-RLKs can be classified into the Groups I, II, and III including Arabidopsis AtWAK-RLKs (Figure 3). Based on these two combined classification system, 29 SlWAK-RLKs in the tomato genome were firstly provided a detail analysis for the characteristics of SlWAK-RLKs protein and gene in tomato, which will expanding our outstanding on their structure, potential biological functions, and regulatory networks in plant kingdom.

A typical WAK-RLK contains an extracellular domain with an EGF-repeat tightly connected to cell wall, a transmembrane domain, and an intracellular serine/threonine protein kinase domain [32]. The extracellular domains of WAK-RLKs can bind to cross-linked pectin, pathogen- and damage-induced pectin fragments, or oligo-galacturonides. The binding of WAKs to these two types of pectin triggers two different types of responses, native pectin interactions can regulate cell expansion during development, and OGs can activate a stress response pathway [49], then the intracellular serine/threonine protein kinase domain as receptor is necessary for these two responses to proceed. In tomato, the structure of SlWAKs are more typical compared with SlWAKLs, 9 out of 11 SlWAKs have these three typical domains (Figure 1). They may have the ability to perceive and recognize the extracellular environment signals directly. For atypical SlWAK-RLKs, only contain intracellular kinase domains, lacking of transmembrane domains or extracellular domains. We suspect such atypical SlWAK-RLKs may cooperate with the typical SlWAK-RLKs to participate in the perception and transmission of extracellular signals, and this hypothesis needs the use biochemistry ligand-receptor binding screen and SlWAK-RLK transgenic plant to further identification.

Tomato is a model plant used to study wounding response [50,51]. However, how SlWAK-RLKs regulating wounding signals and their functions are still unclear. Previous studies have shown that in Arabidopsis and rice, multiple *WAK* members can be induced by wounding, such as *AtWAK1*, *AtWAK2* [10], *AtWAK5*, *AtWAK7* [52,53], *OsWAK1* [53], and *OsWAK25* [54]. However, no research was screen wounding induced WAK-RLKs in genome scope. From our RNA-Seq data, 80% detecting *SlWAK-RLKs* showed significantly up- or down-regulated expression (*p*<0.001) in wounding tomato leaves (Figure 7a.), providing insight that SlWAK-RLKs maybe act as the key RLKs contributing to tomato wound response. WAK-RLK proteins have been reported to be involved in host resistance against various pathogens in plants, including the studies in Arabidopsis [55], rice [56], maize [16], wheat [57]. Whereas the mechanical wounding caused by harsh natural conditions, immature artificial cultivation techniques, insects chewing, and large herbivores biting will open the way to the invasion by microbial pathogens [32]. Therefore, wounding provides nutrients to pathogens and facilitates their entry into the tissue and subsequent infection [53]. During the interaction of plant and pathogen, many plant RLKs, such as FLS2, EFR, and PEPR1 are involved in plant immunity to trigger plant PTI and ETI to anti-pathogens [58]. Wounding induced WAK-RLKs maybe as an injury signal receptor quickly participated in plant defense response paralleled or combined with other RLKs pathway. Recently, Zhang et al. found tomato *SlWAK1* (named *SlWAK4* in our study) coimmunoprecipitated with both pattern recognition receptors Fls2 and Fls3, acting in a complex and playing an important role during later stages of pattern-triggered immunity in the apoplast [19].

Tomato fruits stress responses seem different from the leaf [59,60]. To analyze the difference expression patterns of SlWAK-RLKs between leaf and fruit under mechanical injury, *SlWAKL1* and *SlWAKL6* were selected to analyze time course transcript levels in response to mechanical wounding in mature leaf and mature green fruit. As shown in Figure 7c,d, both genes in leaves were sharply induced after 1 h and the transcription was induced by threefold compared to control. Unlike in leaves, fruits tissue showed 7-fold and 16-fold increase for *SlWAKL1* and *SlWAKL6*, respectively, after injury treatment, and wounding induced their expression sustained a long time than that of the leaves, indicating *SlWAKLs* may play more important role in fruits wounding response. Our cis-acting elements analysis in all *SlWAK-RLKs* promoter regions also reveal that phytohormone responsive cis-acting elements regulated by MeJA, ABA, and SA, widely exist in *SlWAK-RLKs* promoter sequences. Simultaneously, MeJA, ABA, and SA are considered to be some of the primary chemical wound signals [61]. In addition, wound stress-response cis-elements, WUN-motif, exists in almost half of the *SlWAK-RLKs* promoter sequences. These results indicate that the rapid induction of SLWAK-RLKs after wounding may be related to the hormone-mediated transcriptional regulation. Meanwhile, as a receptor-like kinase, finding the co-receptors and substrates of WAK-RLKs and analyzing their roles in wounding response will studied in the following research.

The roles of WAK in plant wounding response are described above. Furthermore, the *WAK-RLKs* have known roles in cell elongation and plant development, such as *AtWAK2* and *AtWAK4* were reported to play roles in cell elongation and cell division [9,10], *OsiWAK1* played roles in pollen viability and germination [62], and *OsDEES1* was involved in rice fertility [63]. However, few studies have shown that *WAK-RLKs* are related to fruit ripening. In our study, expression of various *SlWAK-RLKs* expressed changes during the cell expansion and ripening process of tomato fruits. These results indicate that *WAK*-*RLKs* may participate in the development of tomato fruits, and provide ideas for further research.

## 5. Conclusions

Tomato is an important economic crop and a model plant for fruit ripening and stress response research. In this study, we defined 29 SlWAK-RLK members, and characterized their sequences, structures, cis-elements, expression pattern during fruit development, and under wound stress, which can expand our outstanding on their potential biological functions and regulatory networks in plant kingdom. Stable genetic transformation and virus-induced gene silencing technology have been widely used in tomatoes. Further, we can use these technologies to screen and identify SlWAK-RLKs members involved in fruit development and injury response, which will provide important locus information for breeding tomato cultivars resistant to various stress.

## Figures and Tables

**Table 1:** The characteristics of *SlWAK-RLKs* in tomato.

Name	Gene ID	Chr	AA	MW (kDa)	pI
SlWAK 1	Solyc02g068660	2	701	77.7	7.38
SlWAK 2	Solyc07g007020	7	781	87.4	7.84
SlWAK 3	Solyc09g014710	9	787	87	8.39
SlWAK 4	Solyc09g014720	9	799	88.3	6.13
SlWAK 5	Solyc09g014730	9	757	84.2	6.36
SlWAK 6	Solyc09g014740	9	780	86.7	6.22
SlWAK 7	Solyc09g015230	9	393	44.1	5.74
SlWAK 8	Solyc09g015240	9	764	85.5	6.19
SlWAK 9	Solyc10g076530	10	503	56.4	6.11
SlWAK 10	Solyc10g076550	10	410	45.4	7.06
SlWAK 11	Solyc11g072140	11	765	84.3	5.91
SlWAKL 1	Solyc02g086270	2	775	86	5.65
SlWAKL 2	Solyc02g090110	2	703	77.8	7.73
SlWAKL 3	Solyc03g119240	3	673	75.3	5.55
SlWAKL 4	Solyc04g007390	4	335	33.7	5.39
SlWAKL 5	Solyc04g079710	4	679	75.3	6.82
SlWAKL 6	Solyc05g008930	5	746	84.1	6.77
SlWAKL 7	Solyc05g008940	5	657	74.1	6.65
SlWAKL 8	Solyc05g008950	5	626	70.9	8.38
SlWAKL 9	Solyc05g008960	5	665	75	6.68
SlWAKL 10	Solyc05g008970	5	458	52	6.7
SlWAKL 11	Solyc05g008980	5	631	71	6.19
SlWAKL 12	Solyc05g008990	5	663	74.9	5.81
SlWAKL 13	Solyc05g009010	5	302	35.3	6.6
SlWAKL 14	Solyc05g010530	5	380	42.9	6.31
SlWAKL 15	Solyc09g008640	9	628	70	8.23
SlWAKL 16	Solyc09g011200	9	637	71.3	8.85
SlWAKL 17	Solyc12g036330	12	651	72.9	6.17
SlWAKL 18	Solyc12g088040	12	621	68	5.76

Chr, chromosome; AA, number of amino acid; MW, molecular weight (KDa); pIs, theoretical isoelectric point.

**Table 2:** Putative cis-acting regulatory elements in *SlWAK-RLK* promoters.

	Phytohormone Responsive									Abiotic Stress-Responsive					Development-Related	
Function	Methyl Jasmonate		Abscisic Acid	Salicylic Acid	Gibberellin			Auxin		Light Response		Anaerobic	Wound	Low-Temperature	MYB Related	
Cis-element	CGTCA-motif	TGACG-motif	ABRE	TCA-element	P-box	TATC-box	GARE-motif	TGA-element	AuxR-core	Box 4	TCT-motif	ARE	WUN-motif	LTR	MYB	MBS
***SlWAK1***	**√**	**√**	** **	** **	** **	** **	** **	** **	** **	**√**	**√**	**√**	** **	** **	**√**	**√**
***SlWAK2***	**√**	**√**	** **	**√**	** **	** **	** **	**√**	** **	**√**	** **	**√**	**√**	** **	**√**	** **
***SlWAK3***	**√**	**√**	**√**	** **	** **	** **	**√**	**√**	** **	**√**	**√**	** **	**√**	** **	**√**	**√**
***SlWAK4***	**√**	**√**	**√**	** **	** **	** **	** **	** **	** **	**√**	** **	** **	**√**	** **	**√**	** **
***SlWAK5***	** **	** **	**√**	** **	**√**	** **	** **	**√**	** **	**√**	** **	** **	**√**	** **	** **	** **
***SlWAK6***	**√**	**√**	** **	**√**	**√**	** **	** **	** **	**√**	**√**	** **	** **	**√**	** **	**√**	**√**
***SlWAK7***	**√**	**√**	**√**	**√**	** **	** **	**√**	** **	**√**	** **	**√**	**√**	**√**	** **	**√**	**√**
***SlWAK8***	**√**	**√**	**√**	** **	** **	** **	** **	** **	** **	**√**	**√**	**√**	** **	** **	**√**	**√**
***SlWAK9***	** **	** **	** **	**√**	** **	** **	** **	** **	** **	**√**	**√**	** **	**√**	**√**	** **	** **
***SlWAK10***	** **	** **	** **	**√**	** **	** **	** **	** **	** **	**√**	**√**	** **	** **	**√**	**√**	**√**
***SlWAK11***	** **	** **	**√**	**√**	** **	** **	** **	**√**	** **	**√**	** **	** **	** **	** **	**√**	** **
***SlWAKL1***	**√**	**√**	**√**	**√**	** **	**√**	** **	** **	** **	** **	** **	**√**	** **	** **	**√**	** **
***SlWAKL2***	** **	** **	**√**	**√**	** **	** **	** **	** **	** **	**√**	**√**	**√**	** **	** **	** **	**√**
***SlWAKL3***	** **	** **	**√**	** **	** **	** **	** **	** **	** **	** **	**√**	**√**	** **	**√**	**√**	** **
***SlWAKL4***	**√**	**√**	** **	** **	** **	** **	** **	** **	** **	** **	**√**	** **	** **	** **	** **	**√**
***SlWAKL5***	** **	** **	**√**	**√**	** **	** **	** **	** **	** **	**√**	** **	** **	** **	**√**	**√**	** **
***SlWAKL6***	**√**	**√**	**√**	**√**	**√**	** **	**√**	** **	** **	**√**	** **	**√**	** **	** **	**√**	**√**
***SlWAKL7***	** **	** **	** **	** **	**√**	** **	** **	**√**	**√**	** **	**√**	**√**	** **	**√**	**√**	** **
***SlWAKL8***	** **	** **	** **	**√**	** **	** **	** **	** **	** **	** **	**√**	** **	**√**	** **	**√**	** **
***SlWAKL9***	** **	**√**	** **	**√**	** **	** **	** **	** **	**√**	**√**	**√**	** **	** **	** **	** **	**√**
***SlWAKL10***	**√**	**√**	**√**	** **	** **	**√**	**√**	**√**	**√**	** **	**√**	**√**	**√**	** **	**√**	**√**
***SlWAKL11***	** **	** **	**√**	**√**	** **	** **	** **	** **	** **	**√**	**√**	**√**	** **	** **	**√**	** **
***SlWAKL12***	**√**	**√**	**√**	** **	** **	** **	** **	** **	** **	**√**	** **	**√**	**√**	** **	**√**	** **
***SlWAKL13***	**√**	**√**	** **	** **	** **	** **	** **	** **	** **	**√**	**√**	**√**	** **	** **	** **	**√**
***SlWAKL14***	**√**	**√**	** **	**√**	**√**	** **	** **	**√**	** **	**√**	** **	**√**	**√**	** **	**√**	**√**
***SlWAKL15***	** **	** **	** **	** **	** **	** **	**√**	** **	** **	**√**	** **	** **	**√**	**√**	**√**	** **
***SlWAKL16***	**√**	**√**	**√**	** **	**√**	** **	** **	** **	** **	** **	**√**	**√**	**√**	** **	**√**	** **
***SlWAKL17***	**√**	**√**	**√**	** **	** **	** **	** **	** **	** **	**√**	**√**	**√**	**√**	** **	**√**	** **
***SlWAKL18***	**√**	**√**	** **	** **	**√**	** **	** **	**√**	** **	**√**	**√**	** **	** **	** **	**√**	**√**

**Table 3:** Relative expression analysis of 6 selected *SlWAK-RLKs* in fruits.

	Mature Green	Breaker	Turning	Pink	Light Red	Red
*SlWAK3*	1.0027 ± 0.0746 ^a^	0.5404 ± 0.1003 ^b^	0.5522 ± 0.0262 ^b^	0.3319 ± 0.0090 ^c^	0.2381 ± 0.0240 ^c^	0.2345 ± 0.0186 ^c^
*SlWAK7*	0.0076 ± 0.0002 ^a^	0.0061 ± 0.0007 ^ab^	0.0073 ± 0.0001 ^a^	0.0049 ± 0.00001 ^b^	0.0046 ± 0.0002 ^b^	0.0041 ± 0.0003 ^b^
*SlWAK10*	0.3709 ± 0.0198 ^a^	0.0666 ± 0.0021 ^ad^	0.1161 ± 0.0062 ^c^	0.0781 ± 0.0107 ^b^	0.0375 ± 0.0043 ^d^	0.0632 ± 0.0166 ^bd^
*SlWAKL1*	0.7380 ± 0.1171 ^a^	0.7585 ± 0.0485 ^a^	0.7227 ± 0.1786 ^a^	0.3969 ± 0.0652 ^ab^	0.2154 ± 0.0103 ^b^	0.4637 ± 0.0123 ^b^
*SlWAKL2*	4.6919 ± 0.4591 ^a^	7.3391 ± 1.0864 ^b^	7.5965 ± 1.0379 ^b^	8.4154 ± 0.2462 ^b^	4.3835 ± 0.2620 ^a^	5.0313 ± 0.2608 ^a^
*SlWAKL6*	0.9826 ± 0.1147 ^a^	2.3389 ± 0.1333 ^bc^	3.0420 ± 0.2926 ^c^	3.4917 ± 0.0256 ^c^	1.7636 ± 0.1933 ^ab^	3.2342 ± 0.2895 ^c^

The data is revealed using qRT-PCR in tomato cultivar Micro-Tom. The expression level of SlWAK3 in mature green is set to 1. Data are means ± SD (*n* = 3). Letters in the same line indicate statistically significant difference (*p* < 0.05).

## Supplementary Materials

The following are available online at https://www.mdpi.com/2073-4425/11/10/1186/s1, Figure S1: Sequence alignment of all identified SlWAK-RLK proteins, Figure S2: Analysis of RD-motif, Figure S3: Mechanical Wounding on tomato mature leaves and pericarp, Table S1: The DNA, CDS and AA sequences of SLWAK-RLKs., Table S2: The sequences of RLK-Pelle WAK and RLK-Pelle_WAK_LRK10L-1, Table S3: SlWAK BLASTp output, Table S4: WAKL BLASTp output, Table S5: The AA sequences of AtWAK-RLKs, Table S6: Various cis-acting elements in *SlWAK-RLKs*, Table S7: The SlWAK-RLKs RPKM of various tissues/stages in RNA-seq expression data (Tomato Genome Consortium, 2012), Table S8: The RNA-seq expression data of *SlWAK-RLK* genes in wounded mature leaf for 1 h, Table S9: List of primers used for qRT-PCR analysis, Table S10: HMM search with EGF (PF00008) and pkinase (PF00069) domain.
