# Supplementary material for: Genome-Wide Identification, Classification, Characterization, and Expression Analysis of the Wall-Associated Kinase Family during Fruit Development and under Wound Stress in Tomato (Solanum lycopersicum L.)
Source: Genes (Basel). 2020 Oct 12;11(10):1186. doi: 10.3390/genes11101186 (PMC7650724; doi:10.3390/genes11101186)
Supplement: Supplementary file 1 [file genes-11-01186-s001.zip › Supplementary Files/Supplementary Figure.docx]

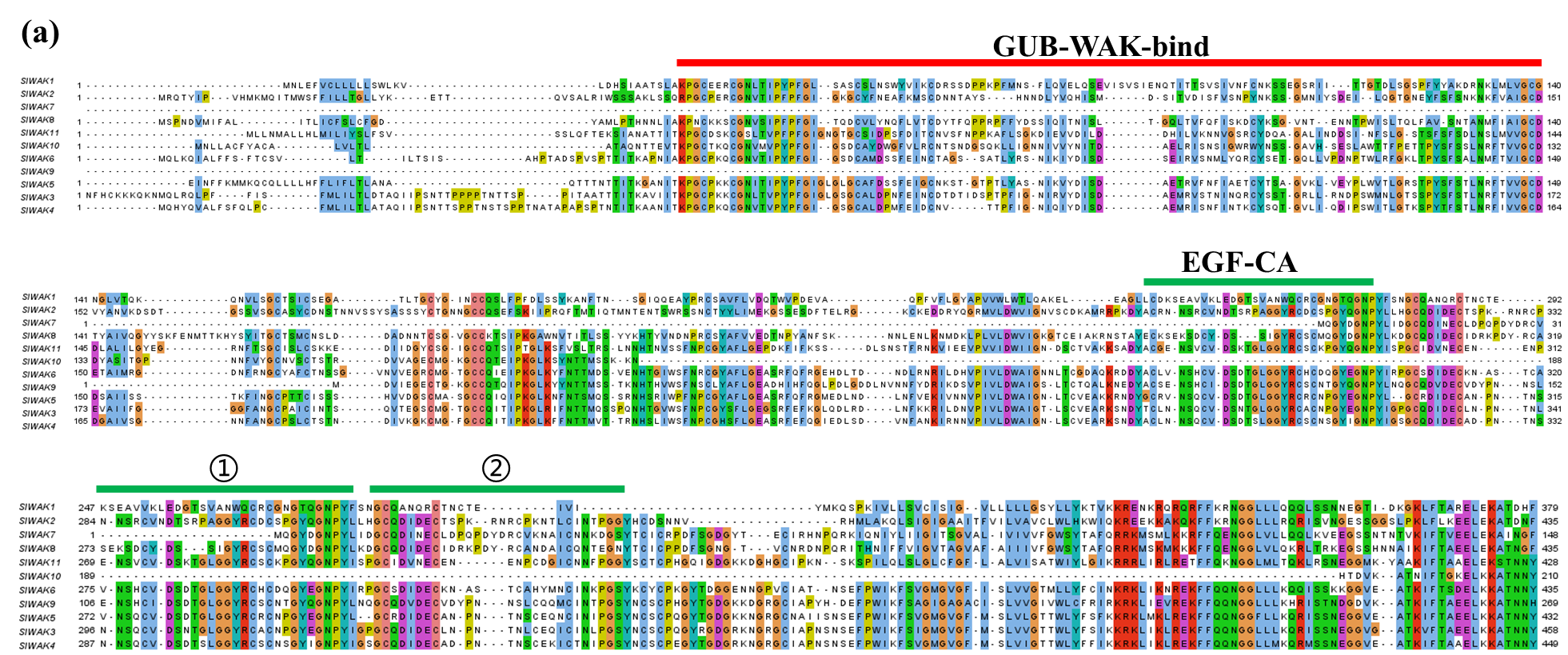


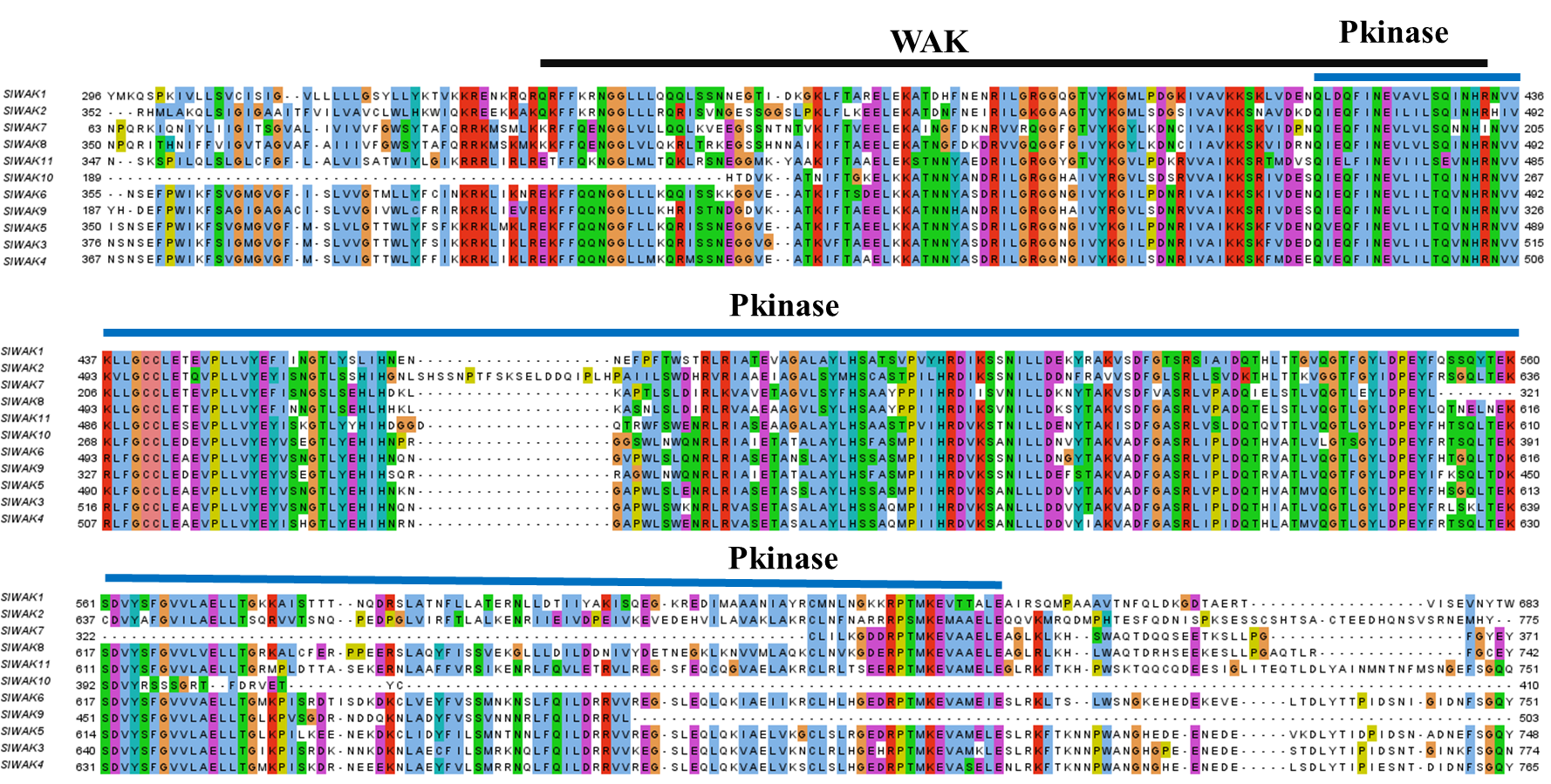


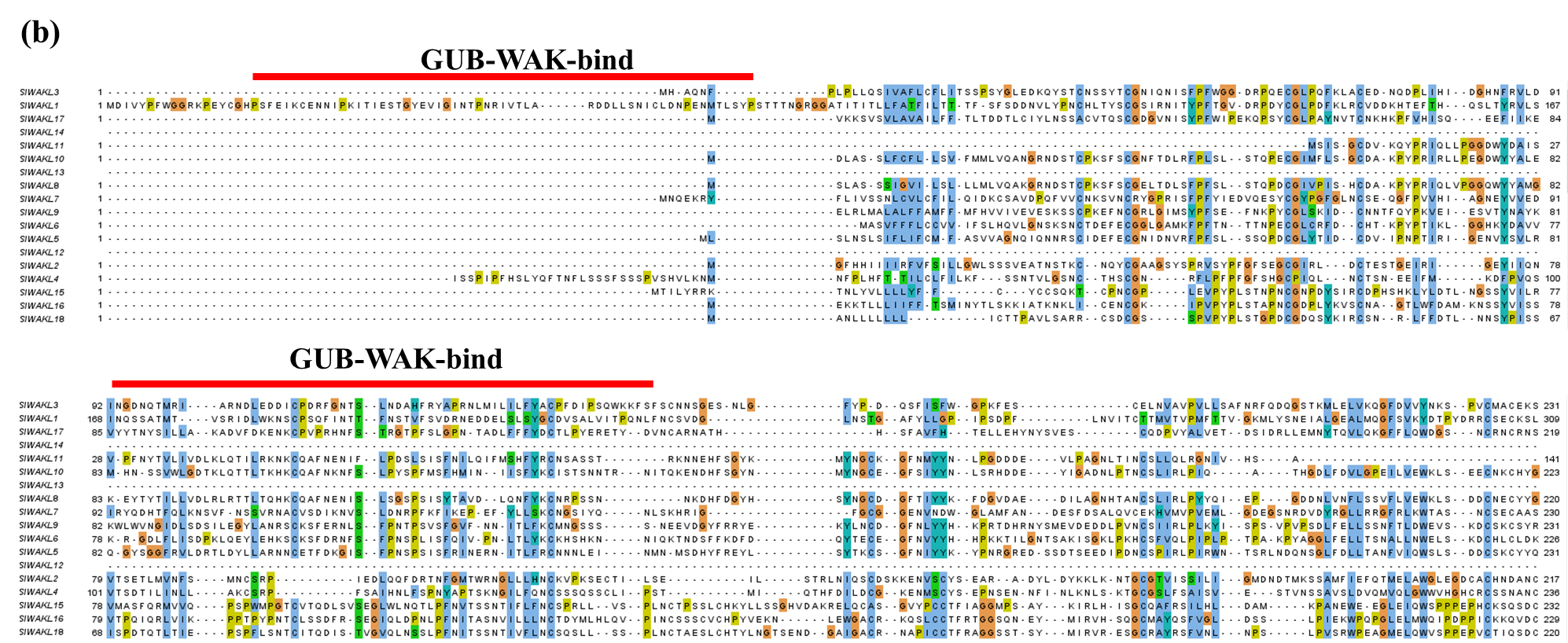


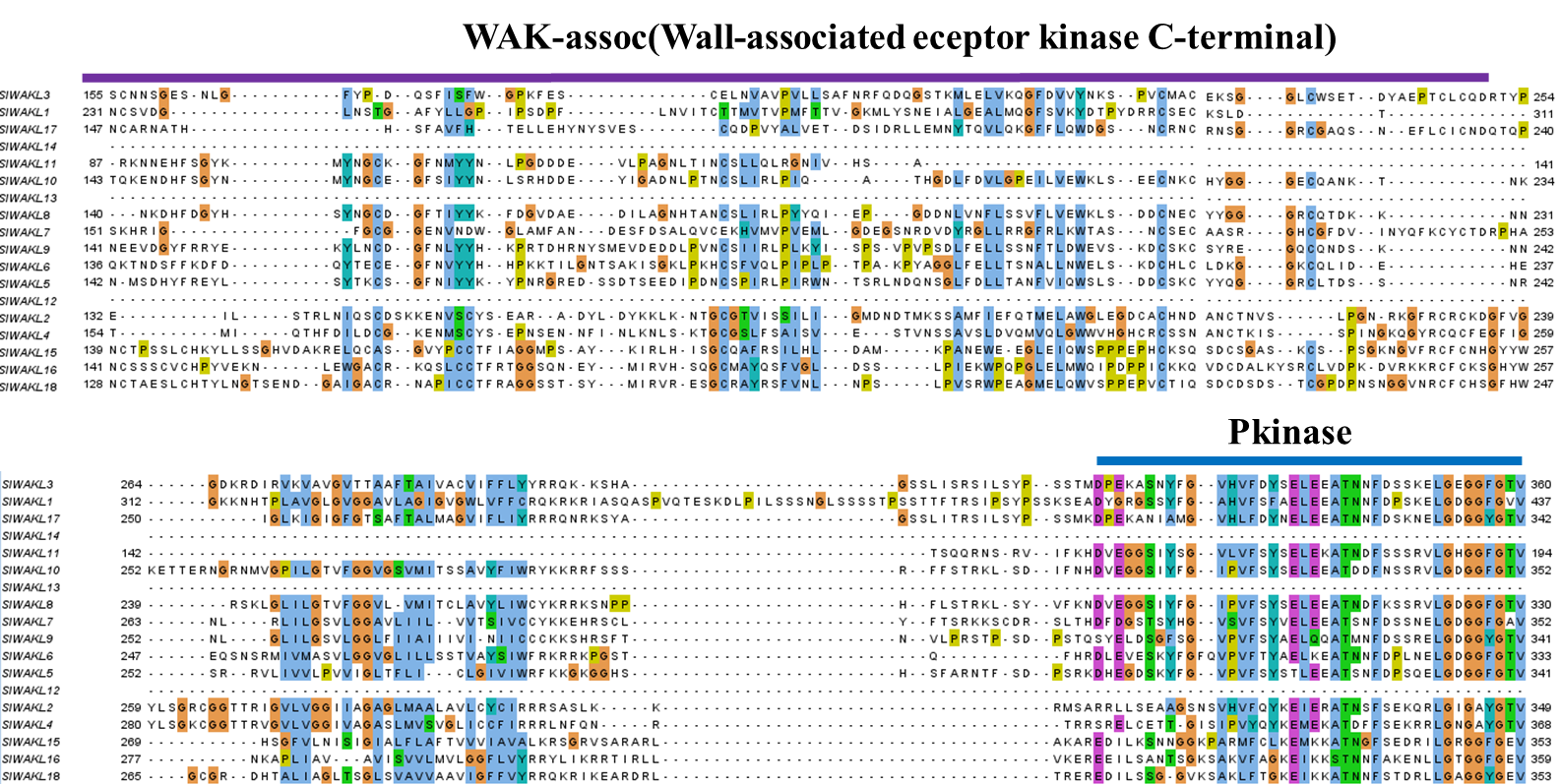


**
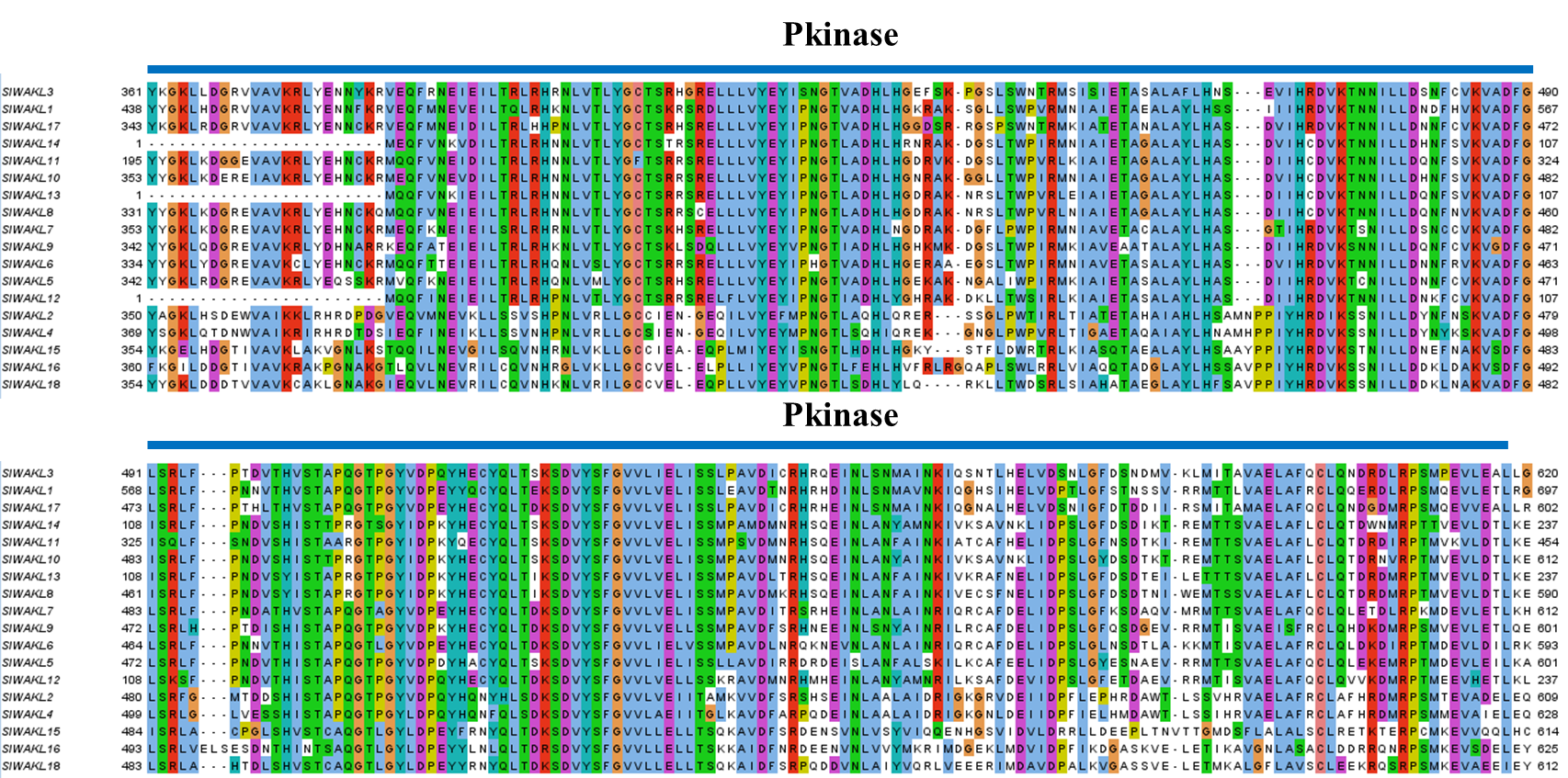
**

**Supplementary Figure 1.** Sequence alignment of all identified SlWAK-RLK proteins. (a) Sequence alignment of all identified SlWAKs. (b) Sequence alignment of all identified SlWAKLs. The conserved amino acid residues are shown in colorful background. The SlWAK-RLK family signature in tomato is underlined with blue, red, green, black, origin, and purple. Red represents GUB-WAK-bind, green represents EGF-CA, black represents WAK, purple represents WAK-assoc, blue represents Pkinase, and orange represents an unpredicted domain.


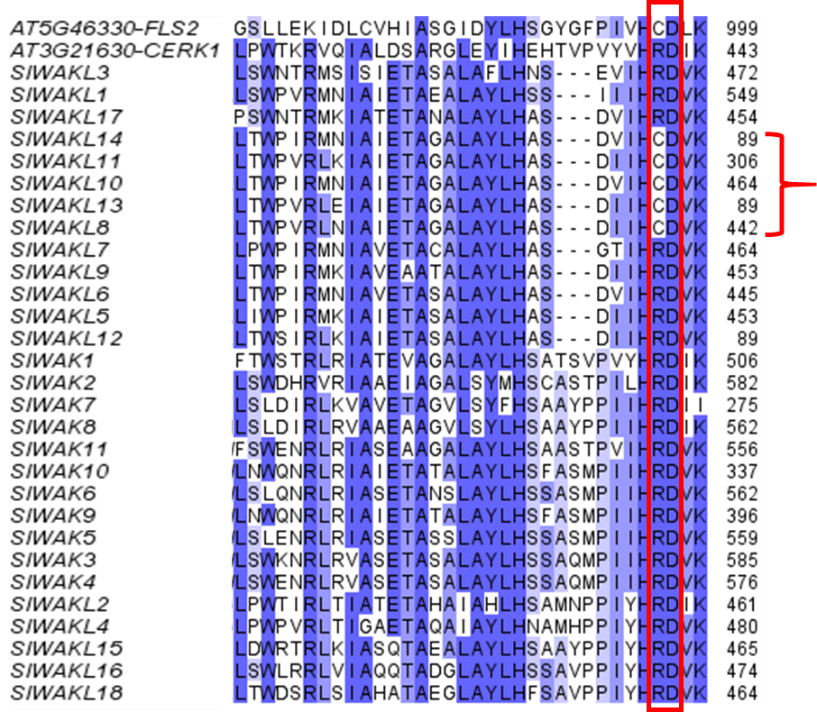


**Supplementary Figure 2.** Analysis of RD-motif. The first protein FLS2 in Arabidopsis is a known Non-RD kinase, and the second CERK1 protein is a known RD kinase. They are used as controls to compare tomato SlWAK-RLK family protein sequences.

**
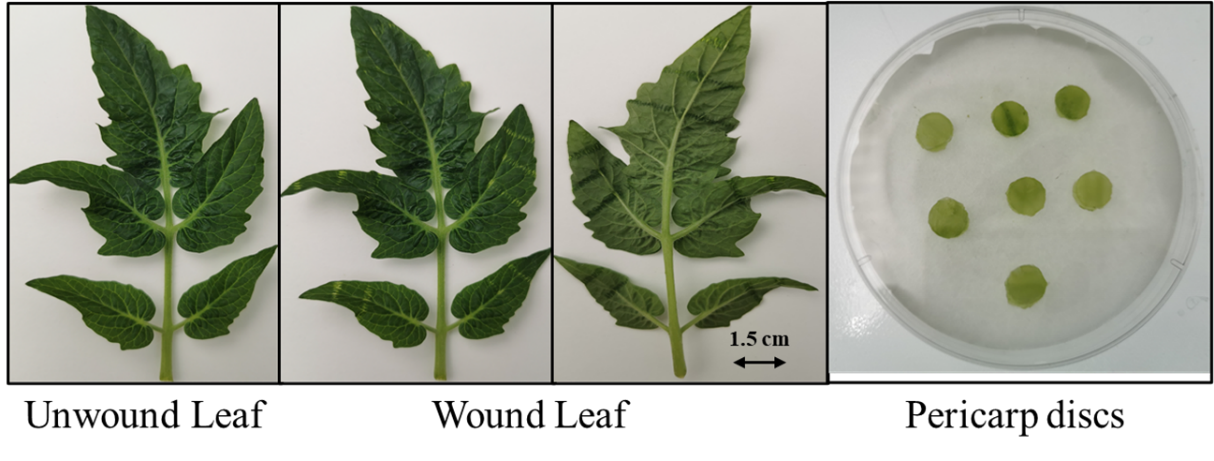
**

**Supplementary Figure 3.** Mechanical Wounding on tomato mature leaves and pericarp
